# Effects of an oral biodegradable device used for 12 weeks on weight reduction, cardiovascular risk factors, satiety, snacking, and meal size

**DOI:** 10.1016/j.obpill.2023.100094

**Published:** 2023-11-13

**Authors:** Haim Shirin, Ian J. Neeland, Donna H. Ryan, Daniel de Luis, Albert Lecube, Zoltan Magos, Yael Kenan, Ruthie Amir, Daniel L. Cohen, Odd Erik Johansen

**Affiliations:** a Shamir Medical Center and Tel-Aviv University, Israel; b Harrington Heart and Vascular Institute, University Hospitals Cleveland Medical Center and Case Western Reserve University School of Medicine, Cleveland, USA; c Pennington Biomedical Research Center, Baton Rouge, USA; d Center of Investigation of Endocrinlogy and Nutrition University of Valladolid Hospital clínico Universitario de Valladolid, Spain; e Endocrinology and Nutrition Department, Arnau de Vilanova University Hospital of Lleida, Biomedical Research Institute of Lleida, Lleida University, Spain; f Aimmune Therapeutics, York, UK; g Epitomee Ltd, Caesarea, Israel; h Nestlé Health Science, Vevey, Switzerland

**Keywords:** Medical device, Weight loss, Observational study, Appetite, Gut-brain axis

## Abstract

**Background:**

The Epitomee Capsule (EC) is an, oral, self‐use, bio-degradable device for weight management, composed of absorbent polymers that self-expands in the stomach (pH-sensitive) and creates a triangular shape, space-occupying super-absorbent gel structure. A recent study reported that 42 % of study completers obtained >5 % weight reduction at 12 weeks. We performed exploratory analyses of this study to evaluate its effect on cardiovascular risk factors and on self-reported satiety, between-meal snacking and meal-size.

**Methods:**

This single-center observational study (Israel) enrolled 78 volunteers, with mean age 41 years, BMI 32.5 kg/m^2^, systolic/diastolic blood pressure (SBP/DBP) 124/77 mmHg. The EC was given in addition to diet and physical activity counseling. Assessments included anthropometrics, BP, lipids, and three questions (translated from Hebrew) scored 1 (not at all) to 5 (very much) for “Do you feel the EC - Q1:helps you to consume less snacks in between meals? Q2:helps you to eat less in the meal?; Q3:is causing an early sense of satiety?”. Changes from baseline were assessed using a mixed model and included all patients with at least one measure. Correlation-analysis between weight-change and PROs used Kendall's tau.

**Result:**

Compared to baseline, at 12 weeks, SBP/DBP were reduced (ΔSBP: −5.5 mmHg, p = 0.0003/ΔDBP: −1.9 mmHg, p = 0.1341), with a larger effect in people with hypertension at baseline (ΔSBP: −13.2 mmHg, p < 0.00001/ΔDBP: −6.1, p = 0.008). Triglyceride-level was also significantly reduced, but not other lipids. Mean scores to Q1-3 were high throughout, with slight decreases (Q1 at W2 3.9 ± 1.1/W12 3.0 ± 1.6; Q2 at W2 3.7 ± 1.1/W12 3.1 ± 1.6; Q3 at W2 3.8 ± 1.2/W12 2.9 ± 1.6). There was a moderate correlation between PROs and weight reduction, although significance was not observed for all weeks.

**Conclusions:**

Exploratory analyses of 12 weeks treatment with EC demonstrated significant reductions in SBP, DBP, and triglycerides. The weight reduction correlated with satiety, less snacking, and reduced meal size.

## 1 Introduction

Obesity, defined as a body mass index (BMI) ≥30 kg/m^2^, continues to increase on a pandemic scale [1], which is projected to impact health due to the detrimental effects of excess weight and adiposity [2]. Epidemiological studies have identified obesity as a risk factor for multiple metabolic and cardiovascular (CV) complications, including arterial hypertension, dyslipidemia, type 2 diabetes mellitus (T2DM), and coronary heart disease [3]. Of importance, research has shown that even a modest reduction in body weight of 3–5% can significantly lower the risk of developing these complications [4], although greater reductions in body weight are associated with larger reductions in risk [5].

The initial steps in weight management involve lifestyle modifications, which include increasing physical activity levels, modifying eating behaviors (including an assessment of how to avoid excess unhealthy snacking and how to obtain meal portion control), and reduced energy diet (e.g., sometimes using meal replacement products or following a particular dietary pattern) [[6], [7], [8]]. However, although this approach can provide weight reduction of 10 % or more for some individuals, behavior changes have proven difficult to adhere to over time [9], and weight regain is common [10]. Several weight reduction pharmacotherapies have now been developed. Each has advantages and disadvantages. Some of the medications approved since 2000, when applied with a lifestyle intervention program, can also provide weight reduction of 10 % on average for most patients, and can improve quality of life [[11], [12], [13]]. In people with T2DM, the GLP-1 receptor agonists may also reduce CV events and mortality [14], and semaglutide recently was indicated to also do so in people with obesity without T2DM [15]. However, the use of these medications can be costly and may result in side effects that lead to discontinuation, and weight regain is frequently seen after their discontinuation [14,16]. Other strategies for weight management include bariatric surgery and intragastric balloons (IGBs) [17,18]. Although both strategies have demonstrated strong efficacy, surgical interventions carry a significantly higher risk than medications, and are also costly and generally considered to be irreversible [17], whereas IGBs are associated with nausea, dehydration, gastric outlet obstruction, retention of foreign body longer than what manufacturer intended, procedural risks during insertion and removal, and unknown long-term weight reduction benefits [18,19].

A relatively new concept of using non-caloric material to stimulate the gut-brain-axis [20], as well as to lower energy density of food without changing macronutrient composition [21], has recently been introduced [22]. This strategy involves the use of non-systemic orally administered capsules. One randomized placebo-controlled study using hydrogel particles taken 30 min pre-meal demonstrated superior weight reduction over placebo [22]. The concept with this particular hydrogel is intended to increase satiety and reduce hunger, although one small mechanistic study on this particular device failed to demonstrate that it influenced appetite [23].

A novel non-caloric medical device, that has been cleared in Europe for use, and that currently is under investigation for potential FDA clearance, is the Epitomee Capsule (EC) [24]. The EC self-expands in the stomach, and is composed of two types of pharmaceutical polymers and bonding materials. One type are superabsorbent polymer miniature particles (particle size of up to 250 μm) that are bonded to create a gel film. The particles are self-expanding and can absorb and retain large volumes of water (approximately 100-times their dry weight), and decrease in volume when ion concentration rises. The other type is a pH sensitive polymer envelope (with a thickness of 20 μm), that remains stable up to pH of 6.5, but above the pH threshold, it disintegrates under weak shear forces. When dry, the entire structure is flat and folded into a standard capsule, whereas after ingestion and absorption of water, the gel particles expand leading to the formation of an elastic structure of triangular shape that resist the contractions of the stomach peristaltic waves, promoting early satiety signaling. The EC consists of 97 % water and 3 % of polymers, and keeps its mechanical rigidity for several hours, until envelope disintegration.

A 12-week single-arm study showed that the EC, combined with diet and physical activity counseling, was associated with a meaningful statistically significant weight reduction from baseline of −3.7 % (intention to treat analysis) and −4.5 % (per protocol analysis), with a favorable safety profile [24]. Herein, we conducted new exploratory analyses of this study to further determine if the weight reduction induced by the EC was linked to improvements in CV risk factors, as well as whether it was associated with effects on pathways related to mechanisms of action such as early satiety, reduced meal size, and decreased snacking.

## 2 Methods

### 2.1 Study design

The design and main results of the study have previously been reported [24]. In brief, this was a prospective, open-label, single center, observational study conducted at the Assaf Harofeh Medical Center in Israel between June 2014 and June 2017. Participants were administered EC capsules (Epitomee Medical, Israel), twice daily, along with low-intensity lifestyle instructions (diet and physical activity plan). Each EC capsule was required to be taken with two cups of water, approximately 30 min before lunch and dinner. During the initial clinic visit, a study dietician created a personalized diet and physical activity plan for each participant based on their daily routine. The participants received lifestyle counseling through phone calls every two weeks, during which the dietician discussed their adherence to the diet and physical activity plan [25]. At each visit, the participant's weight and vital signs were recorded.

The study protocol was approved by the Ethics Committee of the Assaf Harofeh Medical Center (Zerifin, Israel) and was registered on clinicaltrials.gov (NCT03610958).

### 2.2 Patients

The key inclusion criteria for the study were patients with a body mass index (BMI) ranging from 27 to 40 kg/m^2^ between the ages of 21 and 65 years. Additionally, participants needed to be healthy, with normal blood count and chemistry, and willing and able to give informed consent, as well as follow the protocol procedures. The key exclusion criteria consisted of individuals with inflammatory bowel disease, a history of gastrointestinal surgery, and a weight change of more than 5 % within 6 months before the study began. Furthermore, individuals with history or evidence of significant cardiac, renal, neurologic, pulmonary, gastrointestinal, hematologic abnormalities, chronic hepatic disease, or any other disease or symptom that may interfere with the study or confound the results in the judgment of the investigator, were also excluded. The exhaustive list of inclusion and exclusion criterions have been previously published [24].

All patients signed a written informed consent at the time of enrollment. The study was conducted according to the Helsinki Declaration.

### 2.3 Efficacy and safety evaluation

Demographic variables were recorded at the screening visit, whereas clinical parameters and anthropometrics were assessed at baseline and at weeks 2, 4, 6, 8, 10, and 12 as previously described [23]. The absolute weight change was reported for all and by gender. Additionally, the proportion of patients at 12 weeks achieving greater than 3 % and 5 % weight reduction from baseline, as well as those achieving waist circumference reductions greater than 5 cm, were calculated. The CV risk factors systolic blood pressure (SBP) and diastolic blood pressure (DBP) were assessed at the office as resting BP in the morning (non-dominant arm) every two weeks, whereas the laboratory parameters total cholesterol (TC), low-density lipoprotein-cholesterol (LDL-C), high-density lipoprotein-cholesterol (HDL-C), triglycerides (TG), fasting plasma glucose (FPG), and HbA1c, were assessed at baseline, week 6 and week 12. These were analyzed as change from baseline. Additional BP analysis included assessment of the proportion that reached American College of Cardiology (ACC)/American Heart Association (AHA) targets of SBP ≤130 mmHg or DPB <80 mmHg at week 6 and 12 [26]; and by effects on SBP and DBP at week 12 by SBP ≤ or > 130 mmHg at baseline.

Patient-reported outcomes (PROs) about satiety were collected by participants as questionnaires, which were translated from Hebrew, on a weekly basis, onwards from week 1. The PROs were measured on a Likert scale of 1–5, ranging from "not at all" to "very much." The following PROs were assessed: "Do you feel the EC helps you to consume less snacks between meals?" (PRO 1), "Do you feel the EC helps you to eat less in the meal?" (PRO 2), and "Do you feel the EC is causing an early sense of satiety?" (PRO 3). The Hebrew word for satiety, “שובע” is widely understood in Israel.

Safety evaluation included monitoring of any adverse events and laboratory assessment that occurred during the study period.

### 2.4 Statistics

The study involved an exploratory analysis of the complete dataset, which included the patients with at least one parameter measurement. Data changes were analyzed using a mixed model that incorporated both time and subject as fixed and random factors, respectively, and considered all patients with at least one measurement. The mixed model, which is not considered a formal imputation method, is a likelihood-based method and under the MAR (missing at random) assumption it produces unbiased estimates. Assessment of proportions with SBP or DBP above or below 130 mmHg or 80 mmHg, respectively, comparing baseline to week 6 or week 12, were assessed using a McNemar test.

Correlation between PROs and overall weight reduction were analyzed using Kendall's tau (correlation coefficient and p-value), and PROs by tertiles of weight reduction were analyzed descriptively.

Statistical analysis was performed using R (v4.2.2, R Core team 2022) and graphical software was GraphPad Prism 8.0 (GraphPad Software, Inc., San Diego, CA).

A p-value <0.05 was considered statistically significant. As this was an exploratory study, no familywise error rate correction was used.

## 3 Results

**Fig. 1:**
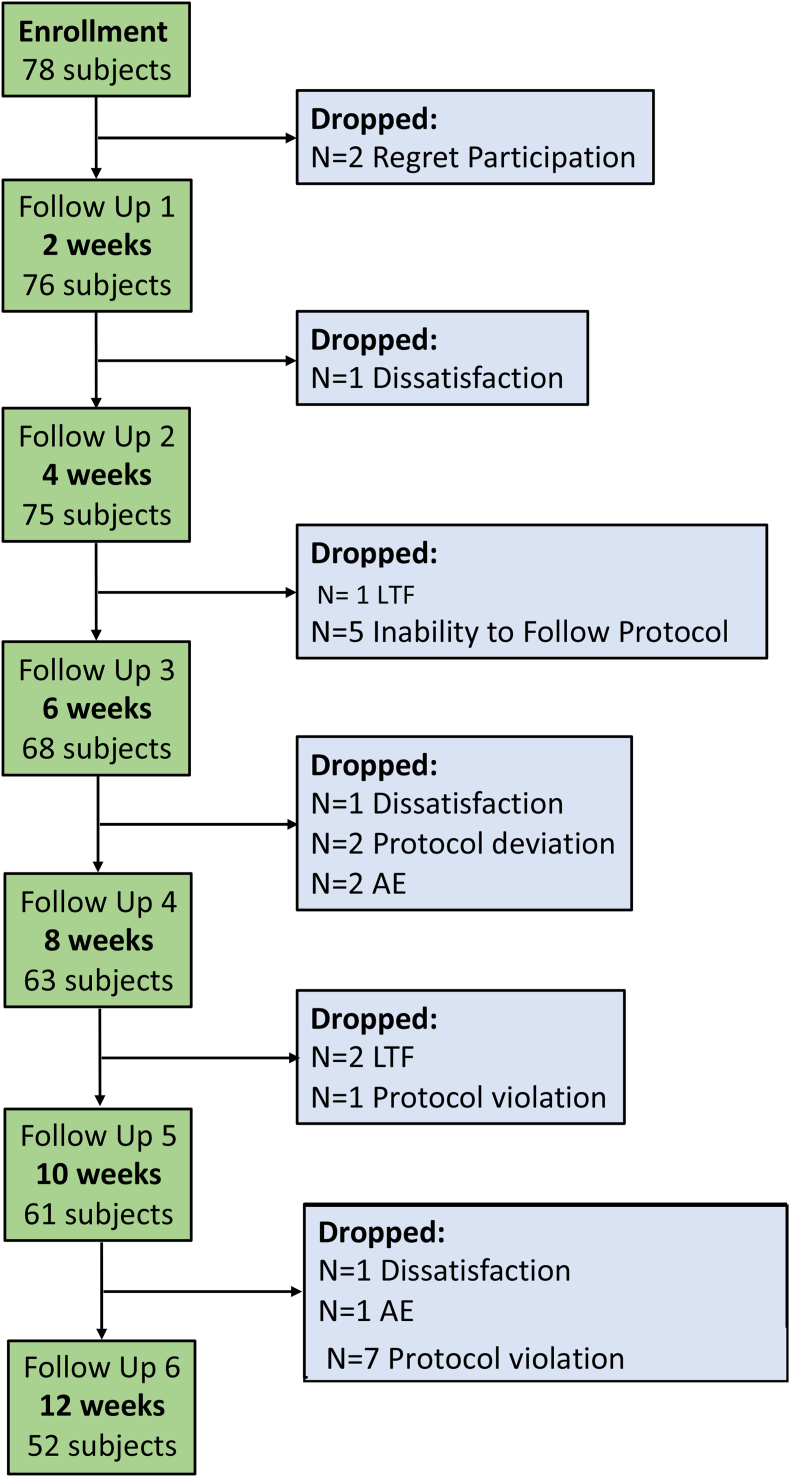
Participant flow from enrollment to 12-weeks follow-up (Consort diagram). Abbreviations: LTF, lost-to-follow; AE, adverse event.

Of 78 individuals who enrolled in the study, 52 (66.7 %) completed the 12-week follow-up (Consort diagram provided in Fig. 1) [24]. The participants had a mean (SD) age of 41 (7) years, and 74.4 % (n = 58) of them were women. At baseline, mean weight was 89.0 (11.4) kg, BMI 32.5 (2.7) kg/m^2^, and waist circumference 105.7 (7.5) cm. Further baseline characteristics, including by gender, are provided in Table 1.

**Table 1:** Baseline characteristics (n = 78). Data are mean (standard deviation) or number (*n*).

	Total	Women	Men
Number of participants, *n*	78	58	20
Age, years	41 (7)	40 (7)	43 (6)
*Race*			
Asian	0 (0)	0 (0)	0 (0)
Black	0 (0)	0 (0)	0 (0)
White	78 (100 %)	58 (100 %)	20 (100 %)
Others	0 (0)	0 (0)	0 (0)
BMI, kg/m2	32.5 (2.7)	32.4 (2.9)	33.0 (2.3)
Waist circumference, cm	105.7 (7.5)	104.2 (7.3)	109.8 (6.5)
Type 2 diabetes mellitus	0 (0)	0 (0)	0 (0)
HbA1c, %	5.3 (0.3)	5.3 (0.3)	5.4 (0.3)
Systolic blood pressure, mmHg	124 (13)	123 (14)	131 (11)
Diastolic blood pressure, mmHg	77 (9)	75 (12)	79 (9)
Fasting total cholesterol, mg/dL	189 (33)	193 (34)	176 (30)
Fasting triglycerides, mg/dL	125 (64)	112 (52)	159 (82)

**Fig. 2:**
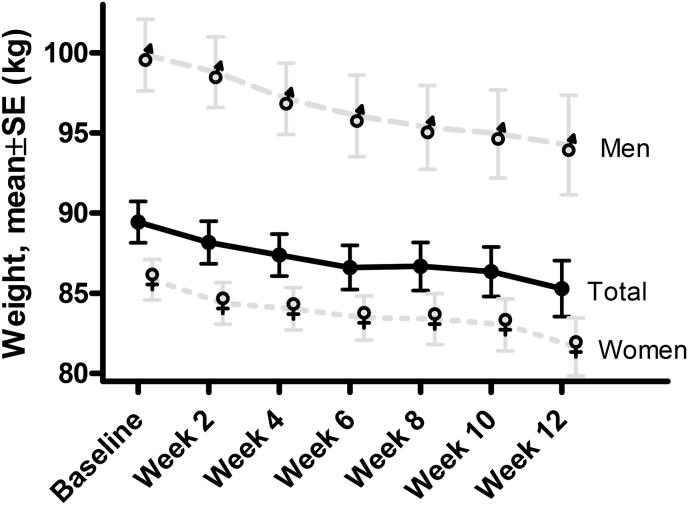
Weight change (overall and by gender) over time.

**Fig. 3:**
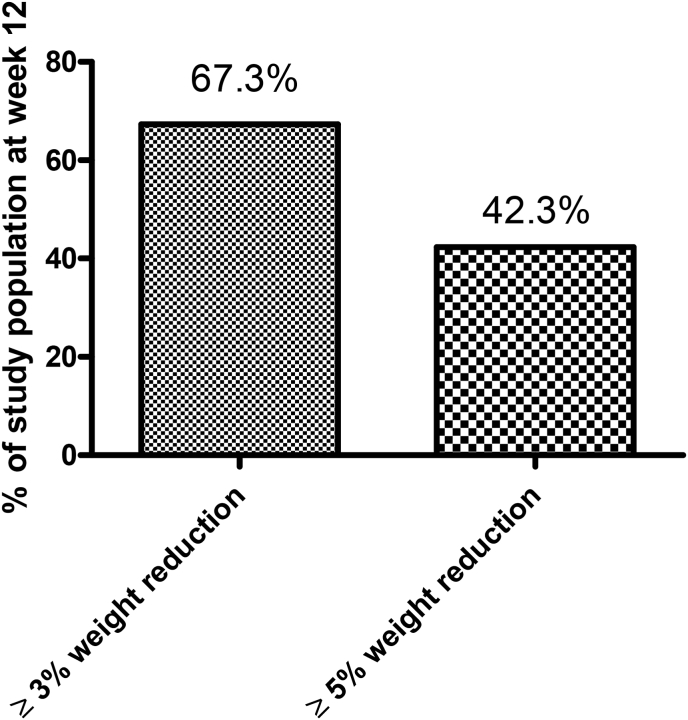
Proportions achieving ≥3 % and ≥5 % weight reduction at week 12.

The observed weight trajectory is presented in Fig. 2, overall and according to gender. The EC was associated with a significant weight reduction at all timepoints as shown in the figure and at week 12 was −4.0 ± 2.6 kg (−4.5 %). The maximum individual weight reduction was −9.4 kg (−10.5 %) (1st tertile weight reduction [−9.4,-3.2] kg, 2nd tertile weight reduction: [−3.2, −1.5] kg, and 3rd tertile weight reduction [−1.5, 2.0] kg) and weight reduction in kg was of a similar magnitude among both sexes across the 12 weeks, despite differing baseline values (women's mean weight at screening visit: 85.4 (9.6) kg vs. men 99.6 (9.8) kg). There was no indication of a plateau of the weight reduction. The proportion of participants that obtained ≥3 % or ≥5 % weight reduction from baseline at week 12 was 67.3 %, and 42.3 %, respectively (Fig. 3), with a corresponding proportion at week 12 that had a reduction in waist circumference >5 cm of 44.2 %.

**Fig. 4:**
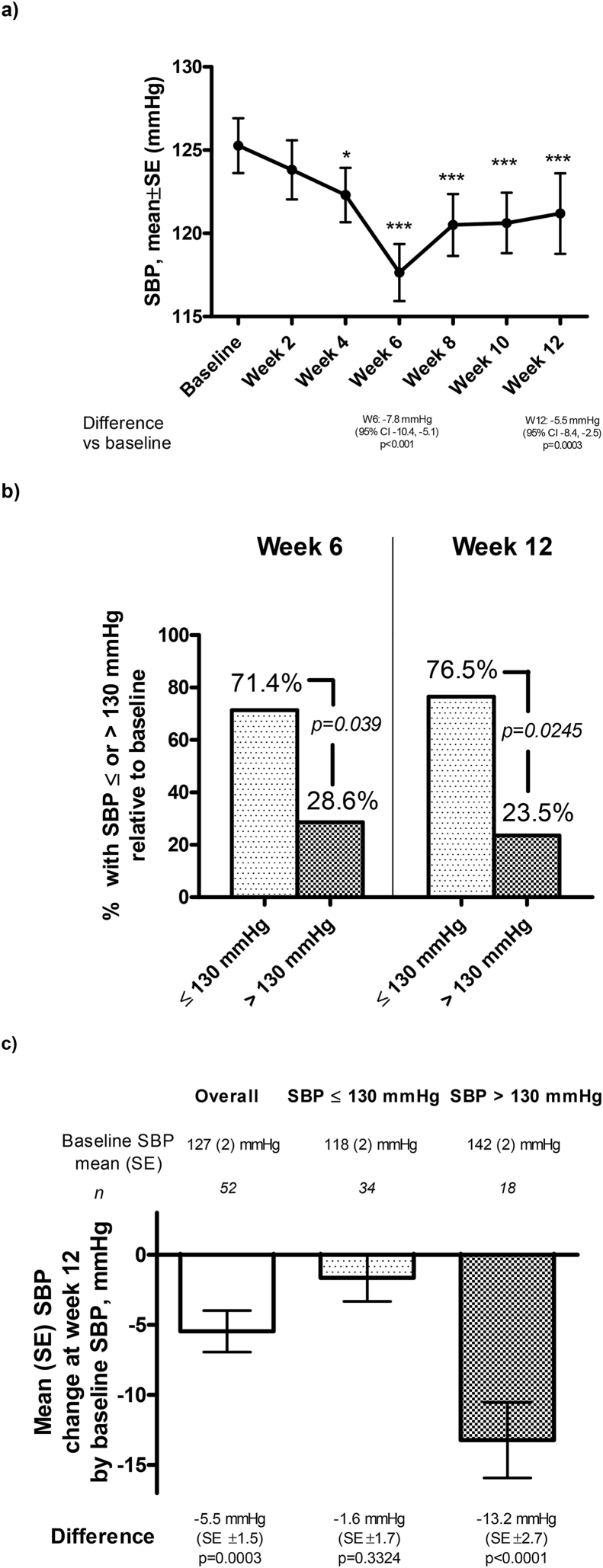
a) Systolic blood pressure over time (difference vs baseline expressed as estimate and 95 ​% confidence interval for estimate with corresponding p-value), b) Proportion of participants with systolic blood pressure ≤130 ​mmHg or ​> ​130 ​mmHg at week 6 and week 12 (difference assessed with McNemar test), c) absolute change in systolic blood pressure by baseline systolic blood pressure ≤130 ​mmHg, or ​> ​130 ​mmHg. ∗: p ​< ​0.05, ∗∗: p ​< ​0.01, ∗∗∗: p ​< ​0.001.

The effects on SBP and DBP are shown in Fig. 4 and Supplementary Fig. 2. As seen (Fig. 4a), there was a reduction in SBP, which appeared to be most pronounced at week 6, and statistical differences relative to baseline were seen at all timepoints onwards from week 4. Of note, a higher proportion of participants obtained a SBP ≤130 mmHg relative to those obtaining a SBP >130 mmHg, at Week 6 (71.4 % vs 28.6 %, p = 0.039) and Week 12 (76.5 % vs 23.5 %, p = 0.0245), respectively (Fig. 4b). An analysis of SBP response at week 12 by baseline SBP ≤130 mmHg or >130 mmHg (Fig. 4c) indicate that the subgroup with SBP >130 mmHg had the largest SBP reduction (SBP_baseline_> 130 mmHg: −13.2 mmHg, p < 0.0001 vs SBP_baseline_≤ 130 mmHg: −1.6 mmHg, p = 0.3324).

The data for DBP, similar to those of SBP, indicate that the most pronounced reduction from baseline was observed at week 6, with statistical significance seen at weeks 6, 8 and 10 (Supplementary Fig. 1a). A higher proportion of participants achieved DBP ≤80 mmHg compared to those obtaining a DBP >80 mmHg at Week 6 (72.2 % vs 27.8 %, p = 0.048) but not at Week 12 (52.9 % vs 47.1 %, p = 0.500) (Supplementary Fig. 1b). An analysis of DBP response at week 12 by baseline SBP ≤130 mmHg or >130 mmHg (Supplementary Fig. 1c) indicate that the subgroup with SBP >130 mmHg had a significant DBP reduction (SBP_baseline_> 130 mmHg: −6.1 mmHg, p = 0.008 vs SBP_baseline_≤ 130 mmHg: 0.3 mmHg, p = 0.8486).

There were no notable effects on lipid parameters (TC, LDL-C, HDL-C) except for a significant reduction in TG levels (baseline mean (SEM) TG: 125 (7) mg/dL) at Week 6 and Week 12 (Week 12 TG: 114 (4) mg/dL (Supplementary Fig. 2 a-d). No discernible effects were observed for HbA1c or FPG (Supplementary Fig. 2 e-f).

**Fig. 5:**
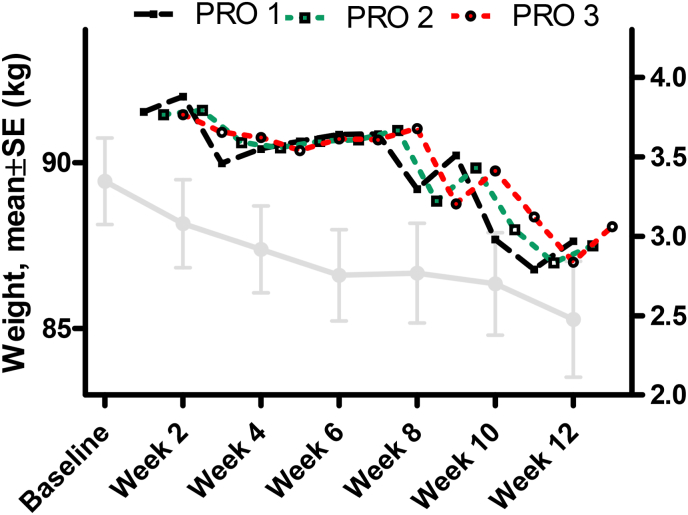
Mean (standard error) weight reduction (kg) over time *versus* mean scores of the patient reported outcomes (PROs) items 1–3′ scores over time.

The mean scores of PROs 1–3 are shown in Fig. 5, and overall, the scores were high throughout weeks 2–12, but showed a decrease at the last two time points (weeks 10 and 12): PRO 1 at W2 3.9 ± 1.1 vs W12 3.0 ± 1.6; PRO-2 at W2 3.7 ± 1.1 vs W12 3.1 ± 1.6; PRO-3 at W2 3.8 ± 1.2 vs W12 2.9 ± 1.6. As a higher score indicates a greater perceived benefit on food intake, this demonstrates a waning of effect; however, there was a consistent pattern of a moderate correlation between PROs and weight reduction at all timepoints, although significance was not observed for all weeks (strongest correlations observed at timepoint W6 for PRO 1 [Kendall's τ coefficient = −0.22, p = 0.026], at W10 for PRO 2 [τ = −0.28, p = 0.01], and PRO 3 [τ = −0.34, p = 0.002]).

**Fig. 6:**
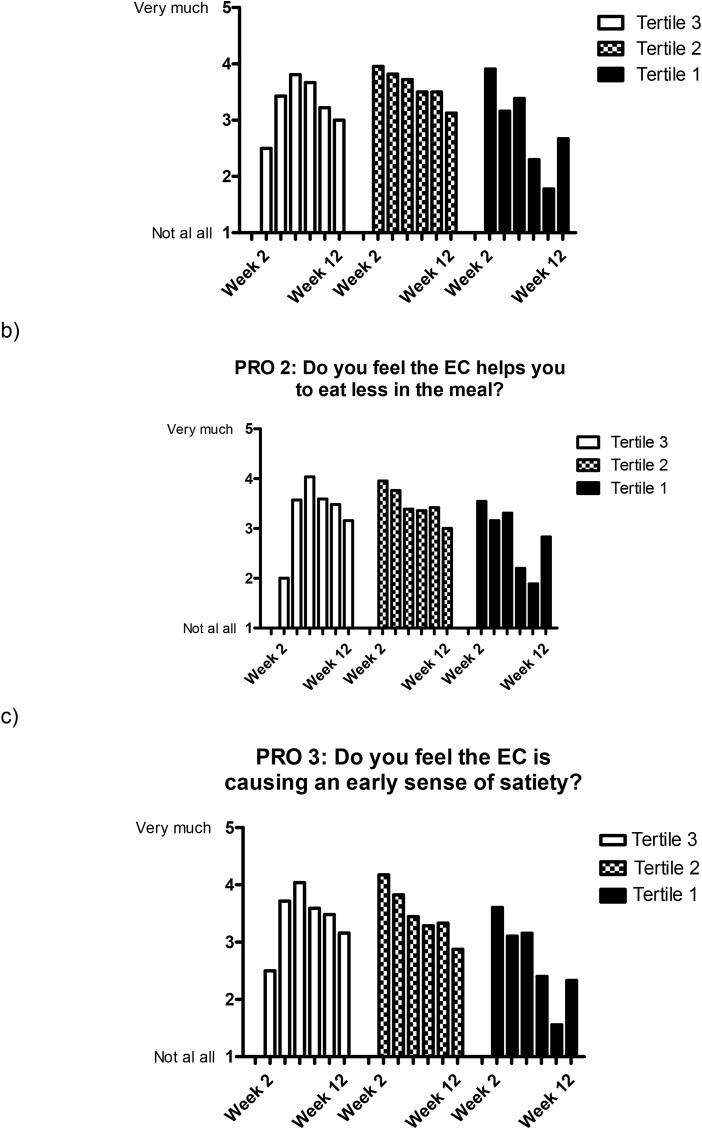
Average scores to (a) PRO 1, (b), PRO 2, and (c) PRO 3 according to tertiles of weight reduction over time (1st tertile weight reduction (−9.4,-3.2] kg, 2nd tertile weight reduction: (−3.2, −1.5] kg, 3rd tertile weight reduction (−1.5, 2.0] kg).

Fig. 6 shows the responses to the PROs 1–3 according to the magnitude of weight reduction, defined by tertiles of weight reduction. As shown, a larger response was observed early on those who obtained the greatest weight reduction [weight reduction ≥ tertile 2 (i.e., >1.5 kg weight reduction)]. However, across all groups, a decrease in PROs occurred with time (Fig. 6).

As previously reported [24], the EC treatment was reported as safe and well tolerated with no reported serious adverse events (SAEs). The most frequent, related or unrelated, adverse events observed were headache, reported by 18.1 % of participants, followed by viral infection (11.1 %), and abdominal discomfort (10.1 %).

## 4 Discussion

The study showed that a 12-week, twice-daily use of the oral biodegradable EC given with low intensity lifestyle instruction resulted in significant weight reduction in both men and women, with associated benefits on CV risk factors and pathways related to mechanisms of action (space-occupying structure), reflected by patient reported improved early satiety, reduced meal size, and decreased snacking. This short-term, uncontrolled, observational study therefore provides preliminary evidence of safety and efficacy, but also has several limitations (open-label, short term exposure, 67 % completers).

The associated effects on CV risk factors associated with modest weight reduction included reductions in waist circumference, where more than 40 % reduced >5 cm, BP improvements, and improvements in TG levels. Of note, significantly greater proportions reached SBP and DBP targets from ACC/AHA of ≤130 and 80 mmHg than those not reaching these [26], respectively, and the BP reductions were particularly pronounced in people who had SBP >130 mmHg at baseline where SBP was significantly reduced by −13.2 mmHg and DBP by −6.1 mmHg. Modulating hypertension through weight reduction is important as people with overweight or obesity often have treatment-resistant hypertension [27,28]. In this context, in light of data showing that not all weight reducing therapies reduce BP [29,30], the present data are therefore reassuring. These observations are also interesting from the perspective of the many signaling mechanisms that connect the gut and the brain in hypertension [31], which include autonomic innervation and vagal signaling, some pathways of which the EC may affect, but which this study is unable to answer, hence further studies are needed.

Effects on other CV risk factors (i.e., TC, LDL-C, HDL-C) or glycemic parameters (FPG or HbA1c) were not prominent in this study, which differs from other studies where a more prominent weight reduction has been reported. However, given that the study population was relatively healthy and young with near-normal baseline values overall and across genders, we speculate that this might have been a reason why improvements in other parameters were not detected.

In the present study, the negative correlations between the PROs and weight change indicate that EC given before meals with two glasses of water and with lifestyle instruction was associated with increased satiety, less snacking, and reduced meal size. Although these findings must be interpreted with caution given the limitations of the study, they are suggestive and clinically relevant as reducing the sensation of appetite and increasing satiety has been demonstrated to be important for weight management [32] and also is involved in different weight reduction approaches [33,34]. Therefore, the satiety sensation experienced by patients treated with EC as prescribed and with low intensity lifestyle counseling as a result of triggering the gut-brain axis [20] may be a significant contributor to their weight reduction. Although there was waning effect on the PROs, suggestive of counter-regulatory mechanisms coming into play or physiological adaptation, there appeard to be no plateauing of the weight reduction. However, since appetite and increased satiety are dimensions that are interrelated, further studies are needed.

There is currently some controversy in the literature whether snacking promotes obesity in adults, however, the consumption of energy-dense snacks and contextual factors related to snacking may contribute to higher energy intake and body weight in adults [35,36]. In this study, patients who reported a reduction in snacking also experienced significant weight reduction during the same period, suggesting a possible association of both conditions. This may also have been influenced by EC which somewhat lowers the energy density of food by creating a transient space-occupying volume in the stomach [20], however, the EC would not be expected to decrease the total caloric content of ingested foods, unless consumption is also reduced, which this study suggest.

Regarding meal size, large portion sizes of commercially available food products have been identified as a likely contributor to the rise in overweight and obesity across the developed world [37]. In addition, there is a consistent body of evidence indicating that manipulating the portion size of a served meal affects acute energy intake during that meal [38]. Therefore, the reported reduction in meal size among the study participants may be one additional factor inducing weight reduction with the EC.

The EC treatment was reported as safe and well tolerated with no reported SAEs. Reported adverse events with EC treatment were mild in severity and transient, which is reassuring.

Again, this study has several limitations. First, this was an open label, single-arm, single-center, prospective study, with no placebo control, in a relatively young, white, healthy population without type 2 diabetes, living with overweight or obesity, which limits the generalizability of the results. While comparing results to the baseline state has some advantages [39], placebo-controlled comparisons are considered the gold-standard. Second, 67 % of all completed the 12 weeks observation, and there were incomplete data collection for some PROs, which may have impacted the statistical power of the study. Additionally, the PROs used were not previously validated questionnaires. Lastly, the present study was not powered to study obesity-related comorbidities, and we did not perform ambulatory BP assessment, but relied on office BP, although the correlation between these is usually acceptable. An ongoing multi-center, double-blind randomized placebo-controlled trial is underway to address these shortcomings in people with overweight and obesity (NCT04222322). The results of this trial will further help inform about the role of the EC in weight management.

## 5 Conclusion

Results from this exploratory analysis of a prospective, open-label, single center study of people with overweight or obesity showed that treatment with EC leads to a significant −4.5 % weight reduction and across gender without SAEs. Specifically, 67 % of enrolled participants achieved a weight reduction of greater than 3 %, with 42 % achieving a weight reduction greater than 5 %. Furthermore, 44 % had a waist circumference reduction greater than 5 cm and this modest weight reduction was associated with statistically significant reductions in SBP, DBP, and TG. Additionally, the weight reduction was related to improvements in PROs for satiety, snacking, and meal size. These results are encouraging as they suggest that EC may potentially represent a new and promising approach for weight management.

## CRediT author statement

The concept and methodology of this submission was by Haim Shirin, Ian J Neeland, Donna H Ryan, Daniel de Luis, Albert Lecube, Yael Kenan, and Odd Erik Johansen. Statistical analysis and data curation was performed by Haim Shirin, Zoltan Magos, and Daniel L. Cohen. First draft writing was by Odd Erik Johansen and all reviewed, edited subsequent versions, and approved the final submission.

## Source of funding

This study was founded by Epitomee Ltd, Israel.

## Ethics review

The study protocol was approved by the Ethics Committee of the Assaf Harofeh Medical Center (Zerifin, Israel) and was registered on clinicaltrials.gov (NCT03610958). All participants provided written informed consent.

## Declaration of artificial intelligence (AI) and AI-assisted technologies in the writing process

During the preparation of this work the authors did not use AI-assisted technologies.

## Declaration of competing interest

HS has received investigator fee from Epitomee, Israel, IJN has received consulting fees/speaking honoraria from Nestlé Health Science, US, 10.13039/100001003Boehringer Ingelheim, Germany, Lilly, US, and AMRA, Sweden, DHR has received investigator fee from Epitomee, Israel. DdL and AL has received investigator fee and consulting fee from Pronokal, a Nestlé Health Science company, Spain, ZM is employed by Aimmune therapeutics, UK, a Nestlé Health Science company. YK and RA are employees and stock-owner of Epitomee, Israel. DLC has nothing to report. OEJ is employed by Nestlé Health Science, Switzerland.
