# Supplementary material for: Effects of an oral biodegradable device used for 12 weeks on weight reduction, cardiovascular risk factors, satiety, snacking, and meal size
Source: Obes Pillars. 2023 Nov 13;8:100094. doi: 10.1016/j.obpill.2023.100094 (PMC10728710; doi:10.1016/j.obpill.2023.100094)
Supplement: Multimedia component 1 [file mmc1.docx]

Supplementary appendix

**Supplementary Figure 1.** a) Diastolic blood pressure over time (difference vs baseline expressed as estimate and 95% confidence interval for estimate with corresponding p-value), b) Proportion of participants with diastolic blood pressure ≤ 80 mmHg or > 80 mmHg at week 6 and week 12 (difference assessed with McNemar test), c) absolute change in diastolic blood pressure by baseline systolic blood pressure ≤ 130 mmHg, or > 130 mmHg. *: p<0.05, **: p<0.01, ***: p<0.001

**a)**

**b)**

**c)**

**
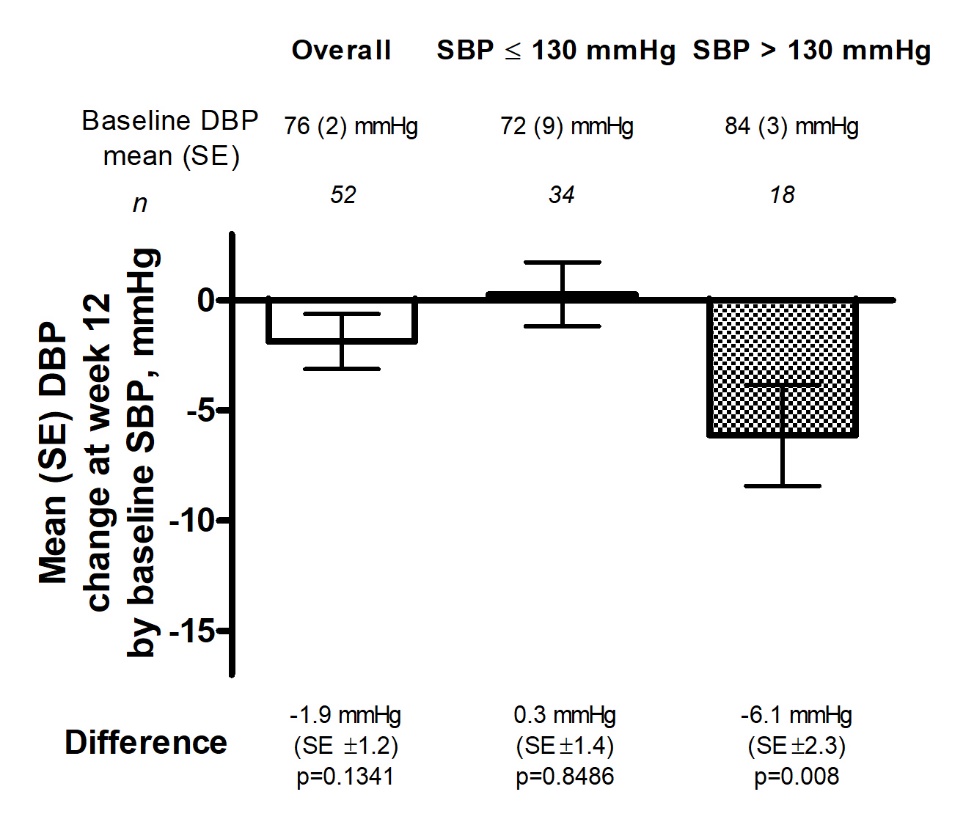
**

**Supplementary Figure 2.** Change from baseline in lipid parameters. (a) Total cholesterol, (b) Low-density lipoprotein (LDL) cholesterol , (c) High density lipoprotein (HDL) cholesterol, (d) Triglycerides, (e) HbA1c, (f) Fasting plasma glucose.

**a)**

**b)**


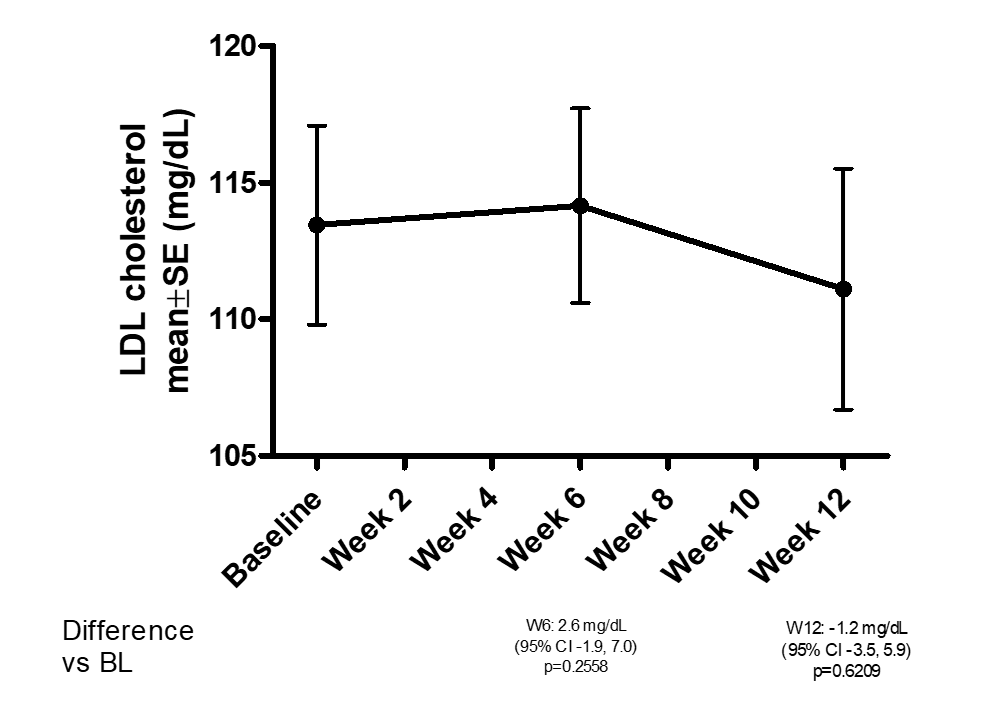


**c)**

**d)**

**e)**

f)
